# Dilated Cardiomyopathy and Hyperthyroidism: A Case Report and Literature Review

**DOI:** 10.7759/cureus.77481

**Published:** 2025-01-15

**Authors:** Oussama Elgharnati, Ikram Damoune, Abdelmajid Chraibi

**Affiliations:** 1 Department of Endocrinology and Metabolic Diseases, Centre Hospitalier Universitaire (CHU) Souss Massa, Agadir, MAR; 2 Laboratory of Kidneys, Endocrinology, Gastroenterology, Neurosciences, and Ethics (REGNE), Faculty of Medicine, Université Ibn Zohr, Agadir, MAR

**Keywords:** dilated cardiomyopathy, heart failure, hyperthyroidism, reversibility, thyrotoxicosis

## Abstract

Dilated cardiomyopathy (DCM) is a rare but potentially reversible complication of hyperthyroidism, often resulting from severe thyrotoxicosis, leading to ventricular dilatation and reduced systolic function. A 45-year-old patient presented with progressive dyspnea and peripheral edema. Laboratory tests and imaging confirmed hyperthyroidism-induced DCM with a reduced ejection fraction (30%). Treatment with carbimazole and propranolol resulted in the normalization of thyroid function and cardiac recovery within three months. Hyperthyroidism can induce cardiac remodeling through genomic and non-genomic mechanisms of thyroid hormones, leading to reversible DCM. Early diagnosis and management are essential to avoid long-term complications, as delayed treatment may result in irreversible myocardial damage. This case highlights the need for multidisciplinary collaboration.

## Introduction

Hyperthyroidism is a common endocrine disorder characterized by the excessive production of thyroid hormones, with an estimated prevalence of 0.5%-1.3% globally [1]. It is well known that hyperthyroidism can cause a wide range of cardiovascular effects, including tachycardia, atrial fibrillation, and, in rare cases, heart failure [2]. While the majority of hyperthyroid patients experience hyperdynamic circulation, some develop severe complications such as dilated cardiomyopathy (DCM), a condition associated with high morbidity and mortality if not promptly treated [3].

Despite the documented association between hyperthyroidism and cardiovascular complications, the exact mechanisms leading to DCM remain incompletely understood. Proposed pathways include direct thyroid hormone-induced myocardial toxicity, chronic tachycardia, and altered calcium homeostasis, which collectively contribute to left ventricular dysfunction and dilation [4]. However, the reversibility of DCM after achieving euthyroidism is not universally observed, and the factors predicting recovery remain unclear.

This case report highlights a rare presentation of hyperthyroid-induced DCM, underscoring the importance of early recognition and timely management to prevent irreversible cardiac damage. Additionally, it addresses the gaps in current understanding by emphasizing the need for further research to elucidate predictive factors for cardiac recovery and optimal treatment strategies.

## Case presentation

A 45-year-old patient presented with progressive dyspnea and lower limb edema. The patient reported no history of heart disease, chronic illness, or regular medication use. On examination, the patient had a heart rate of 120 beats per minute (bpm), a blood pressure of 120/60 mmHg, a temperature of 36.8°C, and an oxygen saturation of 97% on room air. Physical examination revealed jugular venous distension and bilateral pitting edema.

**Figure 1:**
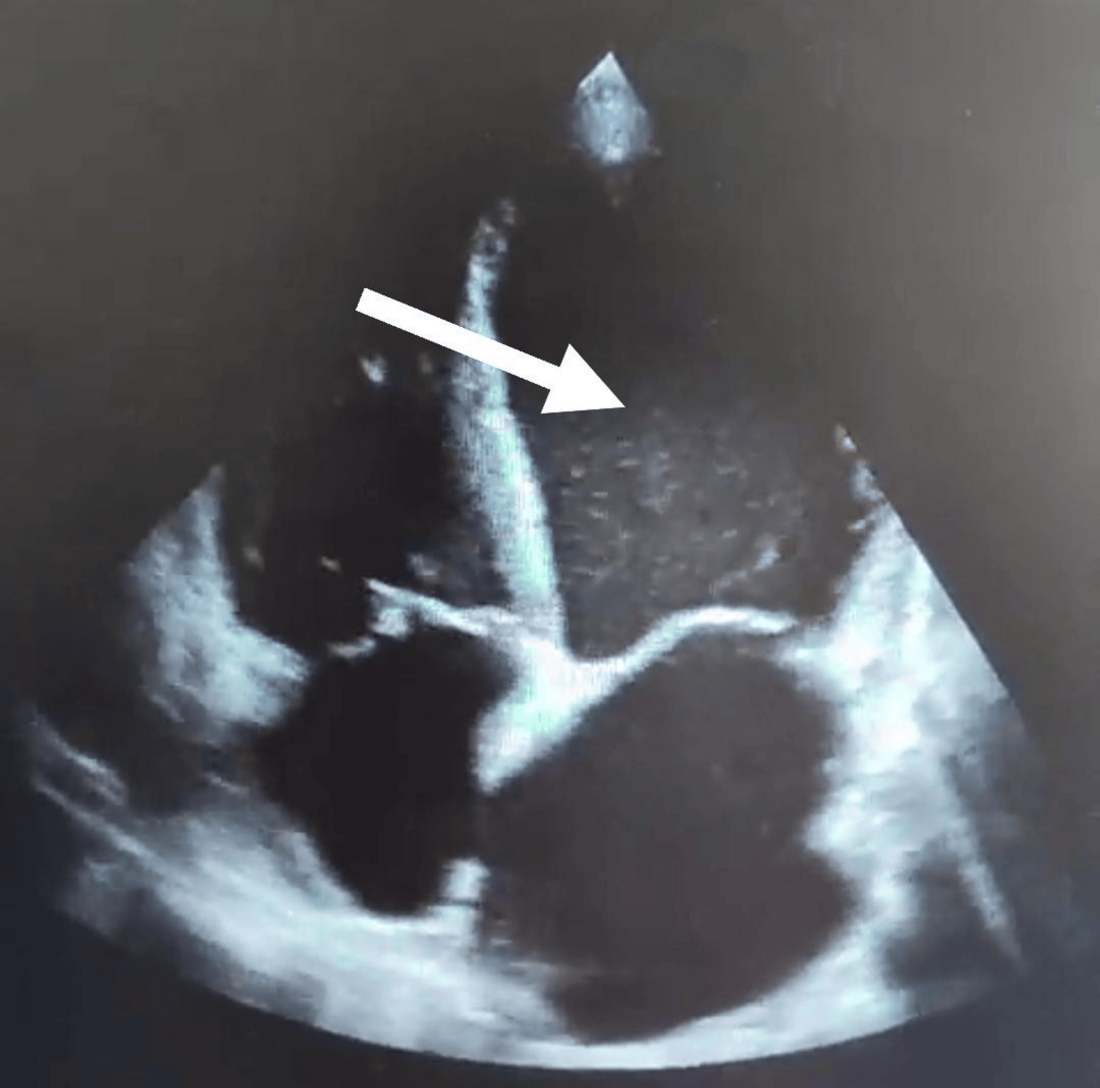
Initial transthoracic echocardiography revealing dilated heart chambers with reduced ejection fraction (30%).

**Figure 2:**
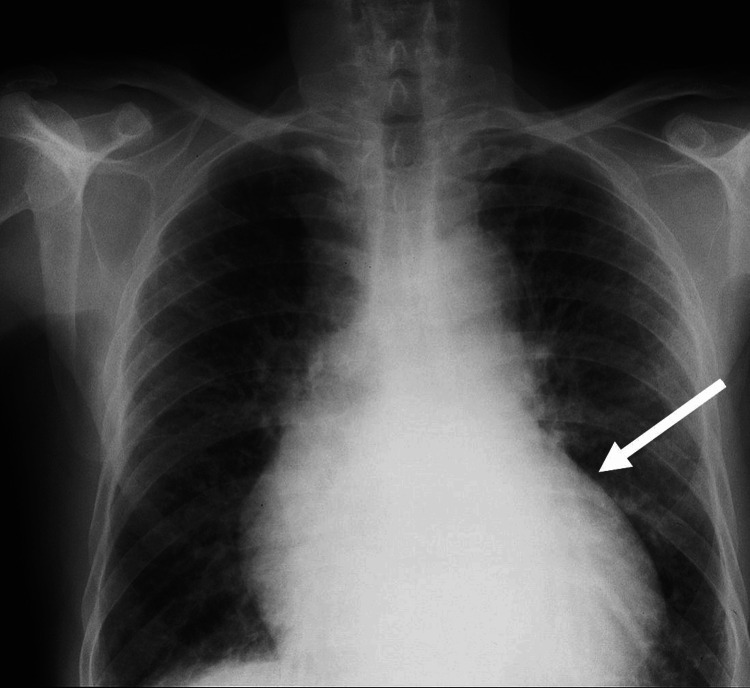
Initial chest X-ray showing cardiomegaly with increased overall dimensions of the heart.

Laboratory tests showed undetectable thyroid-stimulating hormone (TSH) (<0.01 mIU/L) and elevated free T4 (35 pmol/L). Anti-TSH receptor antibodies (TRAK) were positive, confirming Graves' disease. Additional findings included a leukocyte count of 8,500/µL, platelets of 250,000/µL, serum potassium of 4.3 mmol/L, creatinine of 7.2 mg/dL, and glucose of 95 mg/dL. Echocardiography revealed left ventricular dilation with an end-diastolic diameter of 67 mm and a significantly reduced ejection fraction of 30%, consistent with impaired systolic function (Figure 1), consistent with DCM. Chest X-ray showed cardiomegaly, with a cardiothoracic ratio of 62.5% (Figure 2).

Clinical course

**Figure 3:**
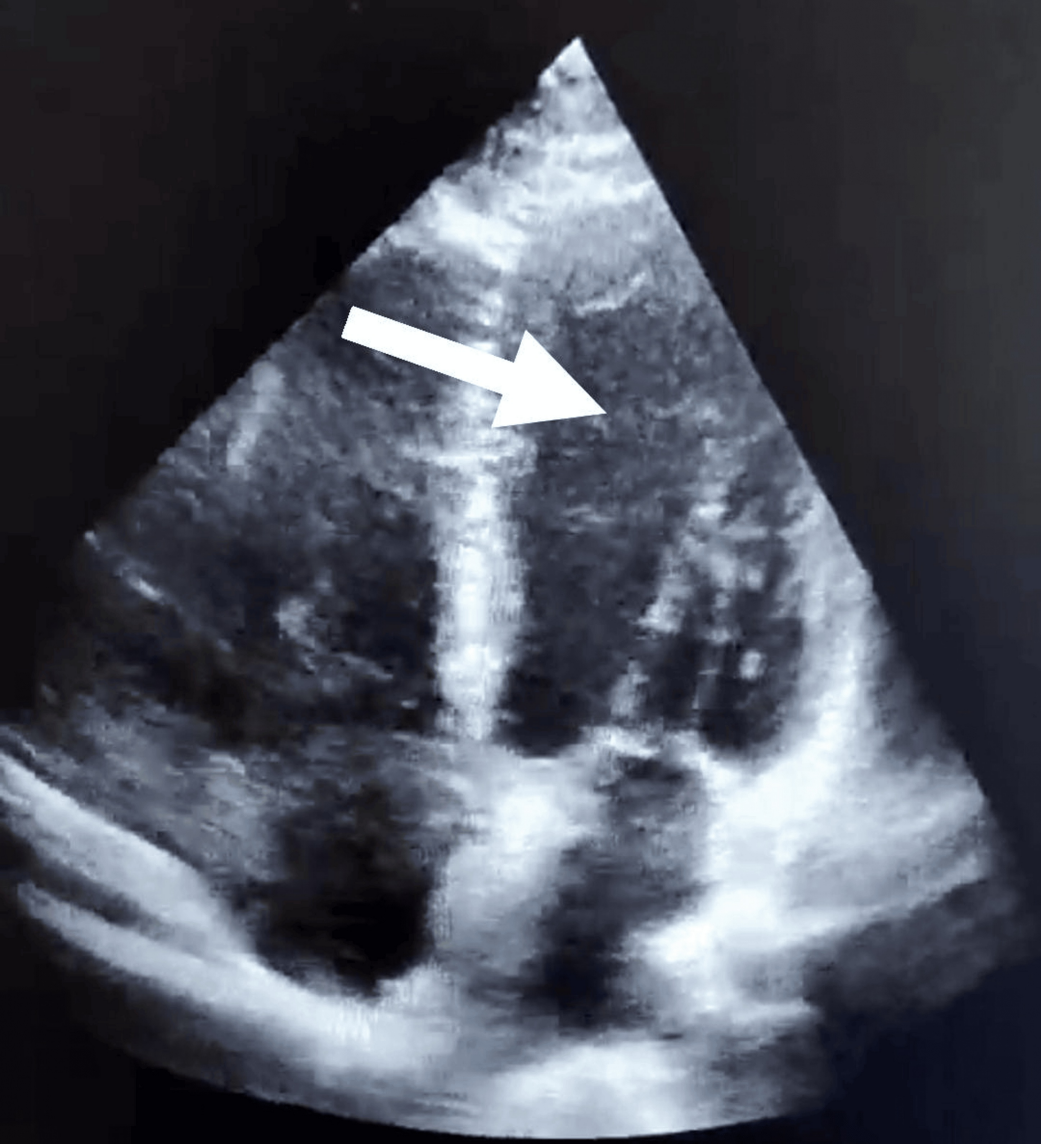
Follow-up transthoracic echocardiography showing the normalization of cardiac dimensions (left ventricular end-diastolic diameter of 52 mm) and improved systolic function (ejection fraction of 60%) with no evidence of hypokinesis.

The patient was treated with carbimazole 20 mg, one tablet daily, combined initially with propranolol 40 mg (quarter tablet, three times daily) during the first two weeks. This regimen led to the normalization of thyroid hormone levels, with a gradual improvement in dyspnea and the resolution of peripheral edema. Three months after initiating treatment, a follow-up transthoracic echocardiogram revealed the normalization of left ventricular ejection fraction to 60% and a significant reduction in cardiac chamber volumes (Figure 3).

## Discussion

Dilated cardiomyopathy secondary to hyperthyroidism is a rare but clinically significant condition. Thyroid hormones influence cardiac function through genomic mechanisms that regulate the transcription of contractile proteins and ion channels and through non-genomic actions affecting myocardial energetics and vascular tone [2,3]. While hyperthyroidism typically induces hyperdynamic circulation, chronic exposure to elevated thyroid hormones can lead to pathological myocardial remodeling, characterized by left ventricular hypertrophy, chamber dilation, and systolic dysfunction [4].

Unlike most forms of DCM, hyperthyroid-induced DCM is often reversible. Studies, including those by Forfar et al., have demonstrated the recovery of cardiac function within weeks to months following the normalization of thyroid hormone levels [5]. In this case, the patient's severe hyperthyroidism and reduced ejection fraction improved significantly with antithyroid therapy and beta-blockers. Osuna et al. reported that over 80% of patients with hyperthyroid DCM achieve full recovery after restoring euthyroidism [6].

However, delayed diagnosis and treatment can lead to irreversible myocardial damage, such as ventricular fibrosis [7]. This case highlights the necessity of early detection and intervention to improve prognosis. Hyperthyroid DCM should always be considered in patients with unexplained heart failure, particularly those without traditional cardiovascular risk factors.

Chronic tachycardia in hyperthyroidism exacerbates cardiac dysfunction and predisposes patients to atrial fibrillation, which occurs in up to 15% of cases [8]. In this case, the electrocardiogram revealed sinus tachycardia at a rate of 120 beats per minute, with evidence of atrial fibrillation with rapid ventricular response. These findings were consistent with the clinical presentation of hyperthyroidism and contributed to the cardiac dysfunction. Atrial fibrillation increases the risk of thromboembolic events, necessitating early anticoagulation in high-risk patients. Chiha et al. emphasized the importance of the timely management of arrhythmias to prevent complications [9].

The American Thyroid Association recommends antithyroid drugs and beta-blockers as the first-line treatment for hyperthyroid DCM [7]. For refractory cases, radioactive iodine therapy or thyroidectomy may be required to achieve long-term control and prevent recurrence. A multidisciplinary approach involving endocrinologists and cardiologists is essential for optimal patient care.

## Conclusions

Hyperthyroidism-related dilated cardiomyopathy (DCM) is a rare but reversible condition if diagnosed and treated early. In contrast to previous reports, this case highlights a particularly rapid recovery in both left ventricular function and dimensions after treatment, which is not always observed in other patients with severe hyperthyroidism. While many studies describe the restoration of cardiac function following the correction of thyroid dysfunction, the extent and speed of recovery in this case are notably pronounced. This underscores the importance of timely, multidisciplinary collaboration between endocrinologists and cardiologists to optimize patient care and prevent long-term complications, especially in patients with severe manifestations of hyperthyroidism.
